# Technological Advancements in Interventional Oncology

**DOI:** 10.3390/diagnostics13020228

**Published:** 2023-01-07

**Authors:** Alessandro Posa, Pierluigi Barbieri, Giulia Mazza, Alessandro Tanzilli, Luigi Natale, Evis Sala, Roberto Iezzi

**Affiliations:** 1 Department of Diagnostic Imaging, Oncologic Radiotherapy and Hematology—A. Gemelli University Hospital Foundation IRCCS, L.go A. Gemelli 8, 00168 Rome, Italy; 2 Istituto di Radiodiagnostica, Università Cattolica del Sacro Cuore, 00168 Rome, Italy

**Keywords:** interventional radiology, interventional oncology, artificial intelligence, digital health, augmented reality, radiomics

## Abstract

Interventional radiology, and particularly interventional oncology, represents one of the medical subspecialties in which technological advancements and innovations play an utterly fundamental role. Artificial intelligence, consisting of big data analysis and feature extrapolation through computational algorithms for disease diagnosis and treatment response evaluation, is nowadays playing an increasingly important role in various healthcare fields and applications, from diagnosis to treatment response prediction. One of the fields which greatly benefits from artificial intelligence is interventional oncology. In addition, digital health, consisting of practical technological applications, can assist healthcare practitioners in their daily activities. This review aims to cover the most useful, established, and interesting artificial intelligence and digital health innovations and updates, to help physicians become more and more involved in their use in clinical practice, particularly in the field of interventional oncology.

## 1. Introduction

Nowadays, physicians are facing an overwhelming amount of complex data, which may impede effective and prompt clinical practice. Artificial intelligence (AI) promises to turn what seems to be a burden of too much information into value. AI represents a very wide field of current technology, also comprising healthcare. Since the 2000s, there has been an exponential growth of AI literature publications from all over the world. Looking at what has been done in the field of radiology in terms of AI, the main areas of interest are represented by neuroradiology, chest, and abdominal imaging, with recent emerging studies on interventional radiology (IR) and, specifically, in interventional oncology (IO) [1]. AI is currently employed in a lot of tasks that might help physicians, and radiologists in particular, in near-future clinical practice tasks, which include automated detection and interpretation of diagnostic imaging findings, comparison and longitudinal analysis of data, patient’s stratification and prognosis evaluation, support to clinical decision-making, and post-processing of images (segmentation, registration, and quantification). Interventional radiologists could take advantage of AI to guide their percutaneous locoregional treatments (e.g., thermal ablation or transarterial radioembolization among the others), and even monitor the outcomes of the treatment (i.e., automatically detecting and segmenting neoplastic liver lesions to analyze treatment response on post-procedural imaging). It is very important to underline the differences between AI and Digital Health (DH), as well as their meaning. AI is based on the construction of complex algorithms driven by a large amount of high quality and very curated data, possibly coming from several centers and pooled together. The future of AI in clinical practice will involve multimodal biomarkers (pathology, genomic, radiomics, and clinical data) to build up complex algorithms and softwares that will provide more efficient and precise applications in everyday clinical practice. Very important features of AI are machine learning (ML) and deep learning (DL). ML is a subset of AI involved with the creation of algorithms which can modify itself without human intervention to produce the desired output by feeding itself through the structured data. Therefore, ML requires structured or labeled data to understand the differences between pathologic and/or physiological conditions and to perform pattern recognition (e.g., images of benign and malignant tumors), to learn the classification, and then to produce the output. ML can provide a fast, reproducible, and reliable tissue characterization (i.e., distinguishing between viable and necrotic tumor, tumor vasculature, healthy liver parenchyma), which is resistant to artifacts and biases. ML algorithms in radiology go through two phases: the first one, called “training”, is characterized by iterative learning to find the best model to classify images (e.g., benign or malignant tumors), whereas the second phase, called “prediction”, consists of applying the best model to classify a new image [2]. DL is a specialized subset of ML which relies on a layered structure of algorithms called “artificial neural network”. Through DL, the machine automatically learns the relevant features or patterns without the need to define features, but in order for that to happen there is a need for a large amount of data (which unfortunately is currently mostly lacking in IO). DL is the ideal natural partner to oncology practitioners which want to perform precision medicine, handling large and heterogeneous datasets for cancer diagnosis (digital pathology and diagnostic imaging), tumor burden, prediction of patient outcomes (e.g., survival, life expectancy, treatment outcome, tumor recurrence), and tailored management. DL can also develop “digital biomarkers”, explain, influence, and predict clinical outcomes. As a confirmation of DL importance, there is the fact that the number of research articles published involving DL in medicine skyrocketed over the past few years [3]. AI is, thus, very different from DH, which includes robotics, augmented reality (AR) and virtual reality (VR), telehealth and telemedicine, as well as practical applications that help physicians in their daily medical activities. Nonetheless, AI and DH are deeply intertwined, and both can help to improve the quality of care in disease diagnosis and patient treatment.

## 2. Radiomics

Radiomics is a science which combines radiology, mathematics and artificial intelligence techniques, using data-characterisation algorithms and mathematical analysis to extract many aspects and traits from radiological imaging; it performs a quantitative approach to diagnostic imaging acquisitions, as opposed to the classical qualitative approach made by physicians [4]. Radiomics evaluates the so-called image biomarkers as digital image texture, consisting of the single pixels and their relationship to the other pixels, as well as intensity and tissue density spatial distribution; these traits, known as radiomic features, may reveal tumoral patterns and characteristics that are not visible to the human eye. The various phases to obtain radiomics features consist of data collection, target lesion segmentation, image biomarkers detection and extraction from image texture, modeling, processing, and validation [5]. The unique imaging characteristics of different disease forms may be helpful in predicting the prognosis and therapeutic response of different types of cancer, thereby offering important information for individualized treatment. The most cutting-edge uses of radiomics are found in radiology and oncology, and therefore, in IO.

### 2.1. Diagnosis

DL systems based on convolutional neural networks (CNNs) have shown potential to revolutionize the process of radiological diagnosis, increasing sensitivity in classifying neoplastic lesions, and giving the radiologist the ability to interpret, check and validate the results [6,7,8]. AI plays an important role in IO, helping physicians to achieve a higher accuracy in the diagnosis of lesions and, thus, to choose the best approach to treatment, personalized for every patient and every neoplastic lesion.

In their study, Hamm et al. tried to develop and validate a DL system based on CNNs, which classifies common hepatic lesions on multi-phasic magnetic resonance imaging (MRI), and compared its performances with those of diagnostic radiologists; test set performance in a single run of random unseen cases showed an average 90% sensitivity and 98% specificity, while the average sensitivity and specificity on these same cases for radiologists was 82.5% and 96.5%, respectively. Results showed a 90% sensitivity for classifying hepatocellular carcinoma (HCC) compared to 60–70% for radiologists [6]. Moreover, the authors have attempted to develop a proof-of-concept “interpretable” DL prototype that justifies the aspects of its predictions from the pre-trained hepatic lesion classifier, identifying and scoring radiological features. This method enables radiologists to interpret elements of decision-making behind classification decisions; this way, clinicians can validate these features by using feature maps or similar interpretability techniques and can check whether the system has accurately identified the lesion’s features in the correct locations [7]. Yasaka et al. in their clinical retrospective study investigated whether different types of liver masses could be differentiated at dynamic contrast agent-enhanced CT by using models based on DL with a CNN. Masses were diagnosed according to five categories (category A, classic HCC; category B, malignant liver tumors other than classic and early HCCs; category C, indeterminate masses or mass-like lesions—including early HCCs and dysplastic nodules—and rare benign liver masses other than hemangiomas and cysts; category D, hemangiomas; and category E, cysts). DL with CNN showed high diagnostic performance; median accuracy of differential diagnosis of liver masses for test data was 0.84 and median area under the receiver operating characteristic (ROC) curve for differentiating categories A-B from C-E was 0.92 [8]. The great promise of AI in interventional oncology is to bring precision medicine at its finest, to the level of the individual patient, through a better understanding and definition of the target lesion. Budai et al. retrospectively constructed a radiomics-based model to diagnose histotypes of renal cell carcinoma (clear-cell versus other histotypes) evaluating the CT scans of 209 patients with renal cell carcinoma, obtaining an accuracy of 78%, sensitivity of 80%, and specificity of 74%, respectively; these results were compared to the ones achieved by an expert radiologist (accuracy of 79%, sensitivity of 84%, and specificity of 69%) [9].

### 2.2. Staging and Outcome Prediction

Staging and outcome prediction is mandatory to address the best treatment for every patient, and this also applies to IO in which locoregional treatments greatly vary based on tumor staging and patient’s outcome, particularly for HCC patients [10].

Most of the current staging systems (e.g., the Tumor, Node, Metastasis (TNM) classification) have limitations in terms of patient prognosis. On the other side, nomograms represent a useful tool in personalized care of oncologic patients, used by an increasing number of cancer centers to improve clinical practice. Nomograms are getting more and more useful in oncological settings as they are capable of estimating the personalized patient risks and prognosis based on disease and subjective characteristics. Even though nomograms’ diffusion dates back to before AI’s, artificial intelligence plays a great role, integrating prognostic and determinant variables, generating a personalized and individualized probability of a patient’s clinical event. Nomograms can be obtained for virtually all types of cancer and can predict various outcomes (prognosis at the time of diagnosis, post-treatment recurrence risk, procedure-specific survival outcomes).

AI-based nomograms with easy-to-use digital interfaces allow for fast and accurate data computation, obtaining clearer and easier to understand prognoses compared to other staging systems, improving the decision-making process. Gupta et al. retrospectively investigated CT images texture to predict grading and survival of 38 patients with suspected gallbladder neoplasm [11]. Multiple authors used radiomics-based models to predict lymph node metastases in various cancer typer (e.g., gastric, breast, bladder, colo-rectal) [12,13,14,15]. AI-based tools can also be used for the staging of the primitive tumor, as well as for prediction of aggressive disease progression [16,17,18,19]. These studies demonstrate how AI outperforms “classical” staging systems, and better determines tumor stage and presence of metastasis, granting a more accurate, personalized and faster treatment choice.

### 2.3. Treatment Response Prediction

ML and DL models can be used in IO practice in predicting response to treatment of cancer patients undergoing locoregional (percutaneous or intra-arterial) therapies. Artificial neural networks that are multilayered or “deep” are the basis of DL. The various neural layers between input and output provide the DL with its plasticity and the ability to define novel patterns of intelligent classification, simulating the workings of the human brain. When compared to a human reader, who can only detect and use a portion of the total big information content of digital images, DL can automatically distinguish the pertinent features from data, allowing it to learn new patterns and determine more complex relationships. This is particularly true in IO where DL-models are capable of integrating multiple patient and tumoral variables, unseen at the human eye, to to guide clinical choices and to predict outcomes of locoregional treatments as transarterial chemoembolization (TACE), radioembolization (TARE), and percutaneous ablation.

Abajian et al. used supervised ML models (logistic regression and random forest) to predict treatment response of patients with liver tumors undergoing TACE. The input multiparametric dataset (age, gender, tumor type, comorbidities, liver function, disease stage, baseline enhancement, wash-out) is elaborated by the ML models to produce an output classification, dividing the patients in treatment responders versus non-responders. The authors obtained an overall accuracy greater than 78% in predicting treatment response to TACE using baseline clinical (e.g., cirrhosis) and diagnostic imaging (e.g., baseline enhancing tumor burden) tumor variables [20]. Morshid et al. evaluated the predictive capabilities of an ML algorithm using clinical parameters and pre-treatment computed tomography imaging features in HCC patients undergoing first-line TACE treatments in terms of time to progression (TTP). The authors classified the patients as TACE-susceptible (in case of high TTP) or TACE-refractory, using diagnostic image features and Barcelona Clinic Liver Cancer (BCLC) stage, obtaining a prediction accuracy rate of 74.2% (versus 62.9% of BCLC stage alone), and potentially aiding HCC patient selection for TACE treatments [21]. Liu et al. built and validated DL radiomics-based algorithms to predict treatment response to first TACE in patients with HCC evaluating pretreatment contrast-enhanced ultrasound (CEUS) acquisitions, with effective and accurate treatment result prediction, also being time- and labor-saving in clinical practice [22]. Peng et al. successfully trained and validated a DL-based model from CT images to predict the response of patients with intermediate-stage HCC undergoing TACE, which had an accuracy of 84.3% [23]. In a retrospective study by Mähringer-Kunz et al., a survival prediction model was developed and validated in order to decide whether repeating or avoiding TACE in HCC patients. The AI-based prediction model was compared to conventional prediction scores and was found to have promising performances in predicting 1-year survival (positive predictive value 87.5%, negative predictive value 68%, sensitivity 77.8%, specificity 81%), significantly outperforming the conventional scoring systems (*p* < 0.001) [24].

Kobe et al. successfully created a ML-model based on texture analysis derived from pre-treatment cone-beam computed tomography (CBCT) in patients undergoing TARE for liver metastases, with high accuracy in treatment response prediction [25]. Ince et al. validated ML-based models with clinical and radiomic features to predict response after TARE. Radiomic features were extracted from pretreatment T1-weighted post-contrast MRI acquisitions, obtained within 3 months before procedure. A total of 1128 features were retrieved among 82 patients (65 responders and 17 nonresponders). In total, 12 features (8 radiomic and 4 clinical) were chosen throughout the selection procedure and were used in the study [26].

Sato et al. developed several ML models to predict tumor recurrence after radiofrequency ablation (RFA) treatment. A total of 1778 patients undergoing RFA for HCC lesions for the first time were included. Tumor number, serum albumin level and des-gamma-carboxyprothrombin level were the most important variables found for the prediction of HCC recurrence [27]. Iezzi et al. retrospectively evaluated contrast-enhanced CT scans in 42 HCC patients (56 lesions) treated with percutaneous ablative techniques (RFA and microwave ablation) to construct a radiomics-based model for early prediction of poor treatment response, obtaining an accuracy of 66%, sensitivity of 85%, specificity of 50%, positive predictive value of 59%, and negative predictive value of 79% [28]. When treating a patient with ablative techniques, it is mandatory to ensure the best lesion coverage as possible, also obtaining a safety ablation margin. The accurate registration of pre-operative diagnostic images to intra- or post-procedural imaging can help in improving needle insertion and treatment results [29]. However, this is not always feasible, particularly due to different patient positioning and variable breathing phases and apnea, as well as due to the difference in image quality and pre-/post-ablation tissue texture. Even though image registration and fusion imaging techniques mostly pertain to digital health, artificial intelligence can bring benefits to this field, overcoming the difficulties related to rigid and non-rigid registration. Wei et al. proposed a DL-based approach to address the registration issues of fusion imaging in US-guided ablations, using a classification network to estimate the US probe angle and then estimating the US plane in the pre-operative CT or MR images through segmentation, with effective results, improving the accuracy of intra-procedural image registration [30].

## 3. AI-Assisted Detection and Segmentation

Computed-aided detection (CAD) is an increasingly utilized tool to perform an adjunctive, second read of diagnostic imaging acquisitions (X-ray, CT, MRI) in order to assist the radiologist in the detection of pathologic lesions and improve their accuracy. Mostly used on chest X-rays and lung CT scans for pulmonary nodules [31,32]. Lee et al. evaluated the efficacy and clinical usefulness of lung nodules’ CAD in patients with colorectal cancer oligometastases to the lung, obtaining good sensitivity and specificity values [31]. Li et al. demonstrated how the recent technological advancements of CAD recognition algorithms increased the accuracy of lung nodule detection up to 99.56%, with a sensitivity of 99.3%, greatly reducing false negatives and missed detections [32]. Ahn et al. determined the usefulness of evaluating breast MRI with a CAD software in the prediction of invasive neoplasm in patients with ductal carcinoma “in situ”, to select patients for sentinel lymph-node biopsy [33]. Takamoto et al. validated a recently developed software of AI-assisted CT-based virtual hepatectomy in patients affected by liver cancer, with a focus on processing time, obtaining reliable and accurate volumetries with a significatively (*p* < 0.001) shorter processing time for AI-assisted reconstructions [34]. AI-assisted CNN-based virtual segmentation can also be useful and time-sparing for volumetries prior to transarterial radioembolization procedures, as demonstrated by Chaichana et al. [35]. The authors developed an automated CNN-based method for target lesion and organ segmentation on Single-Photon Emission Computed Tomography (SPECT) images obtained after 99mTc-labeled Macroaggregated Albumin (MAA) administration, obtaining a time-sparing (about 1 min per patient) and accurate segmentation method. CAD can be an extremely valuable tool for interventional radiologists, as it can provide a faster and more efficient way to detect small or barely visible lesions, leading not only to a correct and early diagnosis but also to a shorter time-to-treatment for patients, which can eventually lead to a better prognosis. As previously stated, CAD may also help IO practitioners to reduce the planning time, as in the case of pre-procedural hepatic volumetric assessment.

## 4. Big Data Issues

The need for AI to access a large amount of data brings forth crucial questions on data handling and privacy. Data collecting systems are different in the USA and Europe. While a data economy already exists in the USA, data regulation in European Countries is different and is based on the General Data Protection Regulation (GDPR) [36]. In Europe, the data privacy and security law is designed to set standards for all sensitive personal data, including healthcare. In the USA a data economy already exists with the Health Insurance Portability and Accountability Act (HIPAA), which was created in 1996, aimed at dealing with protected health information, and includes standards that regulate exchange of protected health information between covered entities (healthcare providers, insurance companies, third party business associates). The USA privacy laws treat big health data in different ways based on how data is created and who is responsible for its custody (physicians, healthcare providers and their associates). However, if on one side HIPAA greatly protects privacy by forbidding the use of personal health information for research without a review board waiver or patient authorization, on the other side the patient’s anonymized health data can easily become identifiable and, moreover, as HIPAA bases its regulations on particular entities and not on the data itself, if a patient gives its data to an uncovered entity HIPAA does not restricts its usage [37]. In Europe, the GDPR defined a broad health data regulation on every personal data related to a patient’s health, regardless of its format, how it was created and collected. The circulation of anonymized datasets between centers in Europe is regulated neither by GDPR nor by any other central law, leading to unclear provisions and impairments in data circulation between centers. These issues need to be taken care of, as AI is becoming increasingly involved in daily routines, both in healthcare and in other life sectors.

## 5. Digital Health

### 5.1. Virtual Reality and Augmented Reality

Digital reality, or extended reality, is an umbrella term which covers all the various technologies that enhance human senses, including platforms that represent cutting-edge technology and that will transform medical training and clinical practice at its most routine levels and at its highest technological point, to drive adoption quality and confidence in performing new procedures with new devices [38]. Nowadays, various forms of digital reality are available and continuously improved:

- Augmented Reality (AR) consists of the addition of digital elements to a live view, basically creating a hybrid of our own reality view and computer-generated objects;
- Virtual Reality (VR) is a completely digital view, where objects and the environment are being replaced by fully digital elements;
- Mixed Reality combines elements of both AR and VR, bringing a technology in which real-world and digital objects simultaneously interact with each other.

One of the most promising digital reality applications is probably navigation, which makes possible many tasks such as layering of medical 2D or 3D images, establishment of the skin entry point, display of the target lesion, visualization of the needle path, identification of structures that should be preserved or are vulnerable in the needle path and also tracking of the distance and angle to target lesions. A traditional CT setup has a monitor directly in front of the scanner, allowing the physician to analyze images and process data on the scanner table with the assistance of a position laser or a laser guide and the sporadic use of in-and-out CT imaging acquisitions to determine the instrument’s position. However, this traditional CT setup lacks real-time feedback information on images, needle position and anatomy, which is where AR may be helpful. MRI can be a challenging modality for interventional procedures since it has longer acquisition time and gantry- as well as magnetic-field-related issues; however, these interventions can be particularly facilitated by AR navigation. A prototypical setup for a navigation system, described in the literature by Fritz et al., uses AR to perform navigation, identification of the anatomic site and of the needle path from the outside, and includes an MRI scanner (or a CT or any hybrid scanner), a dedicated workstation, and a navigation unit. The navigation unit can consist of an MRI-compatible monitor and a semi-transparent mirror to reflect the monitor. Imaging data are acquired and, subsequently, an overlay system allows projection into the mirror and from the mirror into the line of the interventional site; the operator sees both the patient and the MRI (or CT) images or other superimposed information through the semi-transparent mirror. Regardless of where the operator stands, the images are always following, with a laser projected into the skin for determination of the entry point. The display can be mounted with a frame, can be attached to the scanner, or can be freely standing on the other side of the scanner [39]. Another promising application of augmented reality is the teaching of interventional skills: a project where trainers used augmented reality to build up their interventional skills has been carried out. Immersing the learner in a virtual world leads to a higher level of active participation compared to textbooks or online learning modules, because of the student’s increased social, environmental, and personal presence inside the learning activity [40]. In an animal model investigation by Zhang and colleagues using a projectional AR system, it was discovered that operator time for guiding a needle to the target was significantly reduced, and patient-to-image registration error was low [41]. AR with stereotactic navigation includes systems where multiple different electrodes, needles or antennas are inserted in the same patient which still depends upon humans for insertion, but it can plan and track needles path. A result of paper in 301 tumors shows a very precise insertion of needle on particularly difficult to achieve target lesions, with a median lateral and longitudinal error of needle placement of 3 mm [42]. Another paper shows the advantages of stereotactic navigation over manual insertion, demonstrating a primary efficacy rate of 84% in the stereotactic-guidance group versus 75% in the manual-guidance group [43]. Both studies involve complex positions of target lesions and large tumors. A more advanced application of AR involves smart glasses. In this case, images from different modalities are previously loaded into a visor which is synchronized with electromagnetic sensors applied to the patient’s body. This allows a superimposed 3D visualization of the target lesion, of the trajectory line connecting the interventional device to the target, visualization of the organ’s major blood vessels and structures, as well as of the interventional device (needle, electrode), and may be implemented by a target touching phase (i.e., changing color of the target when it is reached by the tip of the needle). In addition, this system has the advantage of changing the modality of guidance (e.g., from CT or MRI to ultrasound room) without losing registration data. It also has to take into account moving organs, for example, pairing with breathing respiratory acts [44]. In a recent study, Long et al. compared smartphone AR, smart glasses AR, and 3D CBCT-guided fluoroscopy for percutaneous needle insertion in a phantom model. The placement error using the 3 systems was similar, but with significantly reduced placement time [45].

Some limitations of AR were described in the recent past, but most of them are nowadays partially or completely resolved, such as:

- The limited field of view (human eyes have a field of view of 200 degrees in horizontal plane and 135 in vertical plane, while head-mounted displays (HMDs) have a field of view of less than 90 degrees;
- Hardware efficacy (however, nowadays even cheaper smartphones meet the minimum requisites);
- Registration mismatch between the real target and the visualized target, or between target and interventional device (which nowadays is less than 5 mm);
- Cybersickness (nausea, headache, dizziness, and vestibular mismatch that can be brought up while using HMDs; however, these symptoms are nowadays dramatically reduced even though subjective to the single physician;
- Time-consuming user-dependent calibration and adjustment with HMDs (no longer needed or greatly decreased nowadays);
- Weight of HMDs.

The main advantages of guidance with AR consist in the significant reduction of radiation dose for procedures performed in the CT room (thanks to the pre-procedural integration of data and minor use of radiographic guidance), system usability both in ultrasound and CT room, for every organ and with any device, high speed and great precision. AR has the potential to change the interaction between imaging formation and clinical practice.

### 5.2. Robotics-Assisted Ablation

Robotics in IO can be of great help as could make treatments available also in countries which lack adequate access to IR specialists. Routinary use of robotics in IO can lead to the reduction of radiation exposure to operators and can increase procedure accuracy in the near future. Robotics systems offering off-plane and multiplanar percutaneous intervention planning, targeting, as well as needle positioning, also using three-dimensional target views, are available for clinical use and can easily support practitioners [46]. Image-guided systems can be useful for spatial positioning and orientation of one or more ablative needle-probes, assisting in manual advancement of the probe (electrode, antenna, etc.) and as intraoperative guidance and post-treatment verification [47]. Robotics-based systems with remote micro- and macro-positioning of the needles can be used for interventions with CBCT, fluoroscopy, and CT in which needle placement is operated by the physician but from a distance [48]. A recent study showed that a table-mounted CT-robot succeeded in reducing microwave ablation needle repositioning attempts and increased accuracy for out-of-plane targets (5.9 mm versus 10.1 mm) but at the cost of longer targeting time compared to freehand targeting (36 min versus 19 min) [49]. CT-guided steerable mini-robots probably represent the most advanced and useful technology in IO nowadays: this robotics-based system is capable of intraprocedural correction of trajectory misalignments during percutaneous procedures [50]. However, many unsolved issues are still under investigation such as cost-effectiveness, standardization, high learning curves, impact on workflow, and so on.

### 5.3. Virtual Multidisciplinary Tumor Board

Integrated multi-disciplinary assessment of every oncological patient undoubtedly leads to better clinical decisions. Virtual multidisciplinary tumor board (v-MDTB) platforms can offer the power to visualize, support, diagnose, and communicate, integrating data from hospital information systems across different clinical domains (such as radiology, pathology, and genomics), thus enabling a consistent, comprehensive, and intuitive view of patient’s relevant information and care path, to facilitate cross-disciplinary collaboration and communication and giving the physicians evidence-based decision tools to promote guidelines adherence and evidence-driven care in personalized medicine. Interactive conferencing, in which a three-dimensional tool can be created, allowing us to virtually discuss how to approach certain percutaneous interventions, can be done with all the physicians together in one room or through teleconferencing [51].

## 6. Conclusions

The aim of every physician should be to use AI in order to obtain the best in clinical practice activities, avoiding redundancies. Given the great help AI can give to every physician in making the work better and faster, implementation of AI techniques and methods in present and future clinical practice should not be feared, as physician’s role will always be fundamental, particularly in case of ethical issues. In the clinical setting, the use of AI promises to lead to a patient-specific IO, with personalized screening and diagnosis, staging and risk assessment, segmentation, fully automated neoplastic tissue compartmentalization, multi-parametric characterization and classification using ML algorithms, treatment choice, as well as tailored post-treatment surveillance and follow-up protocols. AI can provide physicians with “virtual” tissue biopsy, digital pathology, as well as molecular imaging of tissue microenvironment (e.g., hypoxia, tissue metabolism, and immune response). AI can work using integrated multi-modality imaging (e.g., cone-beam CT, positron-emission tomography (PET), and MRI) and fusion imaging, to add powerful and accurate instruments to the interventional radiologist’s toolbox. Treatment prognosis plays an important role in decision making during routine clinical practice, and AI-based models can greatly help in bringing on the best personalized decision for every patient, both for experienced and inexperienced physicians. Nonetheless, physician’s decisions should always be considered, even in case of disagreement with AI suggestions: physician’s knowledge and supervision on AI tasks is still important to grant optimal results. IO can greatly benefit from DH as well as from AI applications, although there is a lack of meaningful and sufficient data to perform training and validation; therefore, clinical trials are welcome to generate more and more data. Governments and hospitals should more and more facilitate the use of AI in common clinical pathways, and interventional radiology can be the stepping stone for this healthcare practice change.

## Data Availability

Not applicable.
